# Supplementary material for: Associations between mental health, blood pressure and the development of hypertension
Source: Nat Commun. 2023 Apr 7;14:1953. doi: 10.1038/s41467-023-37579-6 (PMC10082210; doi:10.1038/s41467-023-37579-6)
Supplement: Supplementary file 1 — Supplementary Information [file 41467_2023_37579_MOESM1_ESM.pdf]

Associations between mental health, blood pressure and the development of hypertension

Supplementary materials

H. Lina Schaare[1,2,3,4], Maria Blöchl[1,5], Deniz Kumral[1,6,7], Marie Uhlig[1], Lorenz Lemcke[8], Sofie L. Valk[2,3,4], Arno Villringer[1,9,10,11]

Affiliations

1 Department of Neurology, Max Planck Institute for Human Cognitive and Brain Sciences, Leipzig, Germany
2 Otto-Hahn-Group Cognitive Neurogenetics, Max Planck Institute for Human Cognitive and Brain Sciences, Leipzig, Germany
3 Institute of Neuroscience and Medicine (INM-7: Brain and Behaviour), Research Centre Jülich, Germany
4 Institute of Systems Neuroscience, Medical Faculty, Heinrich Heine University Düsseldorf, Düsseldorf, Germany
5 Institute for Psychology, Leipzig University, Leipzig, Germany
6 Institute of Psychology, Neuropsychology, University of Freiburg, Freiburg, Germany
7 Institute of Psychology, Clinical Psychology and Psychotherapy Unit, University of Freiburg, Freiburg, Germany
8 Nuclear Magnetic Resonance Unit, Max Planck Institute for Human Cognitive and Brain Sciences, Leipzig, Germany
9 MindBrainBody Institute, Berlin School of Mind and Brain, Berlin, Germany
10 Clinic of Cognitive Neurology, Leipzig University, Leipzig, Germany
11 Charité University Medicine Berlin, Berlin, Germany

Corresponding author: H. Lina Schaare

Max Planck Institute for Human Cognitive and Brain Sciences

Stephanstr. 1A, 04103 Leipzig, Germany

+4934199402220

schaare@cbs.mpg.de

***Supplementary Table 1 – UK Biobank variables used in this study**. We included the following data from the UK Biobank database in the main analyses of this study. Data field IDs with hyperlinks refer to the respective variable that has been used for the analyses.*

Data field ID	Description
Depressive symptoms	
2050	depressed mood
2060	unenthusiasm
2070	tenseness
2080	tiredness
138	Patient Health Questionnaire 9-question version (PHQ-9) items
Well-being	
4526	happiness
4548	health satisfaction
4537	work satisfaction
4559	family satisfaction
4570	friendship satisfaction
4581	financial situation satisfaction
Predictors	
4080	Systolic blood pressure
6150	Hypertension diagnosis
20003	Number of antihypertensive medications
Covariates	
21003	Age
31	Gender
2443	Diabetes status
20002	History of diagnosed angina
20002	History of diagnosed myocardial infarction
20002	History of diagnosed depression
21001	Body-mass index
102	Resting heart rate
20002	History of diagnosed depression
20003	Current intake of antihypertensive medication
20003	Current intake of antidepressive medication
20002	History of severe disease
Imaging-Derived Phenotype (IDP)	
25054	Median z-statistic (in group-defined amygdala activation mask) for faces-shapes contrast
25050	Median z-statistic (in group-defined mask) for faces-shapes contrast
Hospital inpatient records	
41204	Hospital Episode Statistics (HES) database
Additional covariates for sensitivity analyses	
1200	Insomnia
738	Household income
6138	Educational attainment
21000	Racial/ethnic background
54	Assessment centre

Supplementary Table 2 – Diagnoses and details on medication intake for sensitivity analyses. *List of severe disease diagnoses which were considered in sensitivity analyses. Coding refers to the coding as specified in the source data field* *f.20002.0*

Coding	Disease diagnosis
1066	heart/cardiac problem
1073	gestational hypertension/pre-eclampsia
1074	angina
1076	heart failure/pulmonary odema
1077	heart arrhythmia
1078	heart valve problem/heart murmur
1079	cardiomyopathy
1080	pericardial problem
1081	stroke
1082	transient ischaemic attack (tia)
1083	subdural haemorrhage/haematoma
1086	subarachnoid haemorrhage
1093	pulmonary embolism +/- dvt
1123	sleep apnoea
1158	liver failure/cirrhosis
1192	renal/kidney failure
1193	renal failure requiring dialysis
1194	renal failure not requiring dialysis
1196	urinary tract infection/kidney infection
1200	ureteric obstruction/hydronephrosis
1224	thyroid problem (not cancer)
1225	hyperthyroidism/thyrotoxicosis
1229	parathyroid gland problem (not cancer)
1230	parathyroid hyperplasia/adenoma
1232	disorder of adrenal gland
1233	adrenal tumour
1235	hyperaldosteronism/conn's syndrome
1236	phaeochromocytoma
1237	disorder or pituitary gland
1238	pituitary adenoma/tumour
1239	cushings syndrome
1243	psychological/psychiatric problem
1244	infection of nervous system
1245	brain abscess/intracranial abscess
1246	encephalitis
1247	meningitis
1258	chronic/degenerative neurological problem
1259	motor neurone disease
1260	myasthenia gravis
1261	multiple sclerosis
1262	parkinsons disease
1263	dementia/alzheimers/cognitive impairment
1287	anxiety/panic attacks
1288	nervous breakdown
1289	schizophrenia
1290	deliberate self-harm/suicide attempt
1291	mania/bipolar disorder/manic depression
1350	polycystic ovaries/polycystic ovarian syndrome
1371	sarcoidosis
1372	vasculitis
1373	connective tissue disorder
1376	giant cell/temporal arteritis
1377	polymyalgia rheumatica
1378	wegners granulmatosis
1379	microscopic polyarteritis
1380	polyartertis nodosa
1381	systemic lupus erythematosis/sle
1382	sjogren's syndrome/sicca syndrome
1383	dermatopolymyositis
1384	scleroderma/systemic sclerosis
1397	other demyelinating disease (not multiple sclerosis)
1405	other renal/kidney problem
1408	alcohol dependency
1409	opioid dependency
1410	other substance abuse/dependency
1425	cerebral aneurysm
1426	myocarditis
1427	polycystic kidney
1429	acromegaly
1430	hypopituitarism
1431	hyperprolactinaemia
1432	carcinoid syndrome/tumour
1434	other neurological problem
1437	myasthenia gravis
1438	polycythaemia vera
1445	clotting disorder/excessive bleeding
1469	post-traumatic stress disorder
1470	anorexia/bulimia/other eating disorder
1471	atrial fibrillation
1480	dermatomyositis
1481	polymyositis
1483	atrial flutter
1484	wolff parkinson white / wpw syndrome
1485	irregular heart beat
1486	sick sinus syndrome
1487	svt / supraventricular tachycardia
1488	mitral valve prolapse
1489	mitral stenosis
1490	aortic stenosis
1491	brain haemorrhage
1506	primary biliary cirrhosis
1508	jaundice (unknown cause)
1519	kidney nephropathy
1520	iga nephropathy
1546	essential thrombocytosis
1561	raynaud's phenomenon/disease
1583	ischaemic stroke
1584	mitral valve disease

1585	mitral regurgitation / incompetence
1586	aortic valve disease
1587	aortic regurgitation / incompetence
1588	hypertrophic cardiomyopathy (hcm / hocm)
1589	pericarditis
1590	pericardial effusion
1604	alcoholic liver disease / alcoholic cirrhosis
1607	diabetic nephropathy
1608	nephritis
1609	glomerulnephritis
1615	obsessive compulsive disorder (ocd)
1616	insomnia
1659	meningioma / benign meningeal tumour

Supplementary Table 3 – Inclusion/exclusion of previous depression and other BP-altering diseases. *Sensitivity analyses with inclusion and exclusion of subsamples with disease diagnoses at initial assessment. For inclusion/exclusion of depression diagnosis, all multiple linear regression models included age, sex, BMI, resting heart rate, diabetes diagnosis, angina diagnosis, and myocardial infarction diagnosis as covariates. For inclusion/exclusion of any other severe disease diagnosis, all multiple linear regression models included age, sex, BMI, resting heart rate, diabetes diagnosis, lifetime depression diagnosis, and myocardial infarction diagnosis as covariates.*

	**Sub-group**	***N***	**SBP (β)**	**HTN (β)**	**No. anti-HTN (β)**	**Adj. R^2**
**Depressive symptoms**	Depression diagnosis	22,438	-0.052	0.045	n.s.	0.066
	No depression diagnosis	281,333	-0.069	0.046	-0.008	0.052
	Any diagnosis	59,126	-0.055	0.037	0.028	0.149
	No diagnosis	244,645	-0.063	0.050	-0.022	0.119
**Well-being**	Depression diagnosis	9,140	0.059	-0.082	n.s.	0.103
	No depression diagnosis	120,736	0.058	-0.055	n.s.	0.053
	Any diagnosis	24,946	0.046	-0.045	-0.024	0.116
	No diagnosis	104,930	0.058	-0.064	0.021	0.078

Supplementary Table 4 – Medication effects. *Sensitivity analyses exploring medication effects on blood pressure-mental health associations at initial assessment. All multiple linear regression models included age, sex, BMI, resting heart rate, diabetes diagnosis, lifetime depression diagnosis, angina diagnosis, and myocardial infarction diagnosis as covariates.*

	**Sub-group**	**N**	**SBP (β)**	**HTN (β)**	**No. anti-HTN (β)**	**Adj. R^2**
**Depressive symptoms**	Antidepressants	28,722	-0.042	0.045	n.s.	0.110
	No antidepressants	275,049	-0.064	0.046	-0.013	0.065

	Any medication	252,073	-0.057	0.027	-	0.138
	No medication	51,698	-0.080	0.068	-	0.063
**Well-being**	Antidepressants	12,046	0.040	-0.068	n.s.	0.120
	No antidepressants	117,830	0.056	-0.057	0.009	0.059
	Any medication	105,800	0.053	-0.041	-	0.094
	No medication	24,076	0.067	-0.076	-	0.056

Supplementary Table 5 – Multiple Imputation of missing data*. Comparison of cross-sectional models derived from analyses using datasets with listwise exclusion of missing data (complete cases) and multiple imputation (imputed). All multiple linear regression models included age, sex, BMI, resting heart rate, diabetes diagnosis, lifetime depression diagnosis, angina diagnosis, and myocardial infarction diagnosis as covariates.*

		**Outcome**					
		**Depressive symptoms**			**Well-being**		
	**Predictor**	*Estimate*	*SE*	*p*	*Estimate*	*SE*	*p*
**Complete cases**	SBP	-0.002	0.000	0.000	0.002	0.000	0.000
	HTN	0.049	0.003	0.000	-0.070	0.004	0.000
	No. anti-HTN	-0.005	0.001	0.001	0.007	0.002	0.005
**Imputed**	SBP	-0.002	0.000	0.000	0.002	0.000	0.000
	HTN	0.083	0.002	0.000	-0.094	0.004	0.000
	No. anti-HTN	-0.002	0.001	0.043	0.006	0.002	0.015

Supplementary Table 6 – Assessment of survival bias. *Sample characteristics at baseline assessment for total sample and non-surviving sub-sample.*

	**Died after baseline (N=20,442)**	**Overall (N=502,494)**
**Gender**		
Female	8116 (39.7%)	273378 (54.4%)
Male	12326 (60.3%)	229115 (45.6%)
Missing	0 (0%)	1 (0.0%)
**Age (years)**		
Mean (SD)	61.4 (6.55)	56.5 (8.10)
Median [Min, Max]	63.0 [40.0, 70.0]	58.0 [37.0, 73.0]

Missing	0 (0%)	1 (0.0%)
**Townsend deprivation index**		
Mean (SD)	-0.658 (3.42)	-1.29 (3.10)
Median [Min, Max]	-1.55 [-6.26, 10.9]	-2.14 [-6.26, 11.0]
Missing	20 (0.1%)	624 (0.1%)
**Systolic blood pressure (mmHg)**		
Mean (SD)	142 (19.9)	138 (18.6)
Median [Min, Max]	141 [76.5, 254]	136 [65.0, 254]
Missing	2307 (11.3%)	45540 (9.1%)
**Diastolic blood pressure (mmHg)**		
Mean (SD)	82.1 (10.8)	82.2 (10.1)
Median [Min, Max]	82.0 [36.5, 133]	82.0 [36.5, 148]
Missing	2305 (11.3%)	45528 (9.1%)
**Heart rate (beats/min)**		
Mean (SD)	72.1 (12.8)	69.3 (11.2)
Median [Min, Max]	71.0 [33.5, 148]	68.5 [30.5, 173]
Missing	2305 (11.3%)	45528 (9.1%)
**BMI (kg/m2)**		
Mean (SD)	28.2 (5.44)	27.4 (4.80)
Median [Min, Max]	27.4 [12.8, 74.7]	26.7 [12.1, 74.7]
Missing	328 (1.6%)	3105 (0.6%)
**Diabetes**		
Prefer not to answer	21 (0.1%)	404 (0.1%)
Do not know	81 (0.4%)	1280 (0.3%)
No	17592 (86.1%)	473479 (94.2%)
Yes	2700 (13.2%)	26399 (5.3%)
Missing	48 (0.2%)	932 (0.2%)
**Angina**		
No diagnosed angina or unknown	15513 (75.9%)	358910 (71.4%)
Diagnosed angina	1883 (9.2%)	16117 (3.2%)
Missing	3046 (14.9%)	127467 (25.4%)
**Heart attack**		
No diagnosed heart attack or unknown	15762 (77.1%)	363524 (72.3%)
Diagnosed heart attack	1634 (8.0%)	11503 (2.3%)
Missing	3046 (14.9%)	127467 (25.4%)
**Lifetime depression**		
No diagnosed depression or unknown	16028 (78.4%)	346919 (69.0%)
Diagnosed depression	1368 (6.7%)	28108 (5.6%)
Missing	3046 (14.9%)	127467 (25.4%)
**No. antihypertensive medication**		
Mean (SD)	0.852 (1.25)	0.403 (0.864)
Median [Min, Max]	0 [0, 9.00]	0 [0, 9.00]
**No. antidepressant medication**		
Mean (SD)	0.120 (0.346)	0.0784 (0.281)
Median [Min, Max]	0 [0, 3.00]	0 [0, 5.00]
**Current depressive symptoms**		
Mean (SD)	1.48 (0.599)	1.40 (0.528)
Median [Min, Max]	1.25 [1.00, 4.00]	1.25 [1.00, 4.00]
Missing	2662 (13.0%)	53563 (10.7%)

**Well-being**		
Mean (SD)	4.37 (0.620)	4.46 (0.579)
Median [Min, Max]	4.40 [1.00, 6.00]	4.50 [1.00, 6.00]
Missing	14704 (71.9%)	330042 (65.7%)
**Diagnosed hypertension**		
No diagnosed HTN or unknown	12070 (59.0%)	365819 (72.8%)
Diagnosed HTN	8324 (40.7%)	135745 (27.0%)
Missing	48 (0.2%)	930 (0.2%)

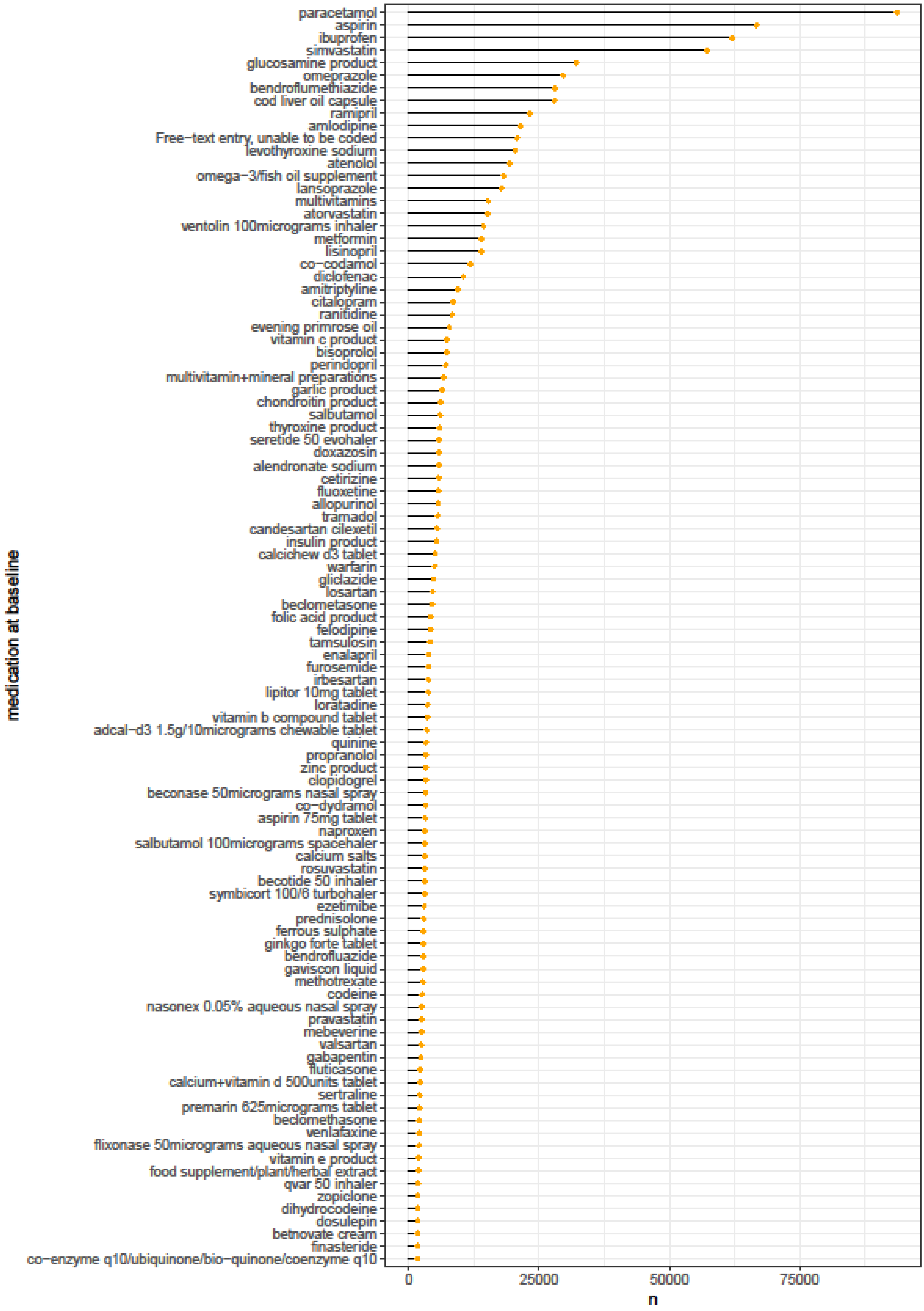

Supplementary Figure 1 - Medication intake frequency of UK Biobank participants at baseline (100 most frequently reported drugs depicted).

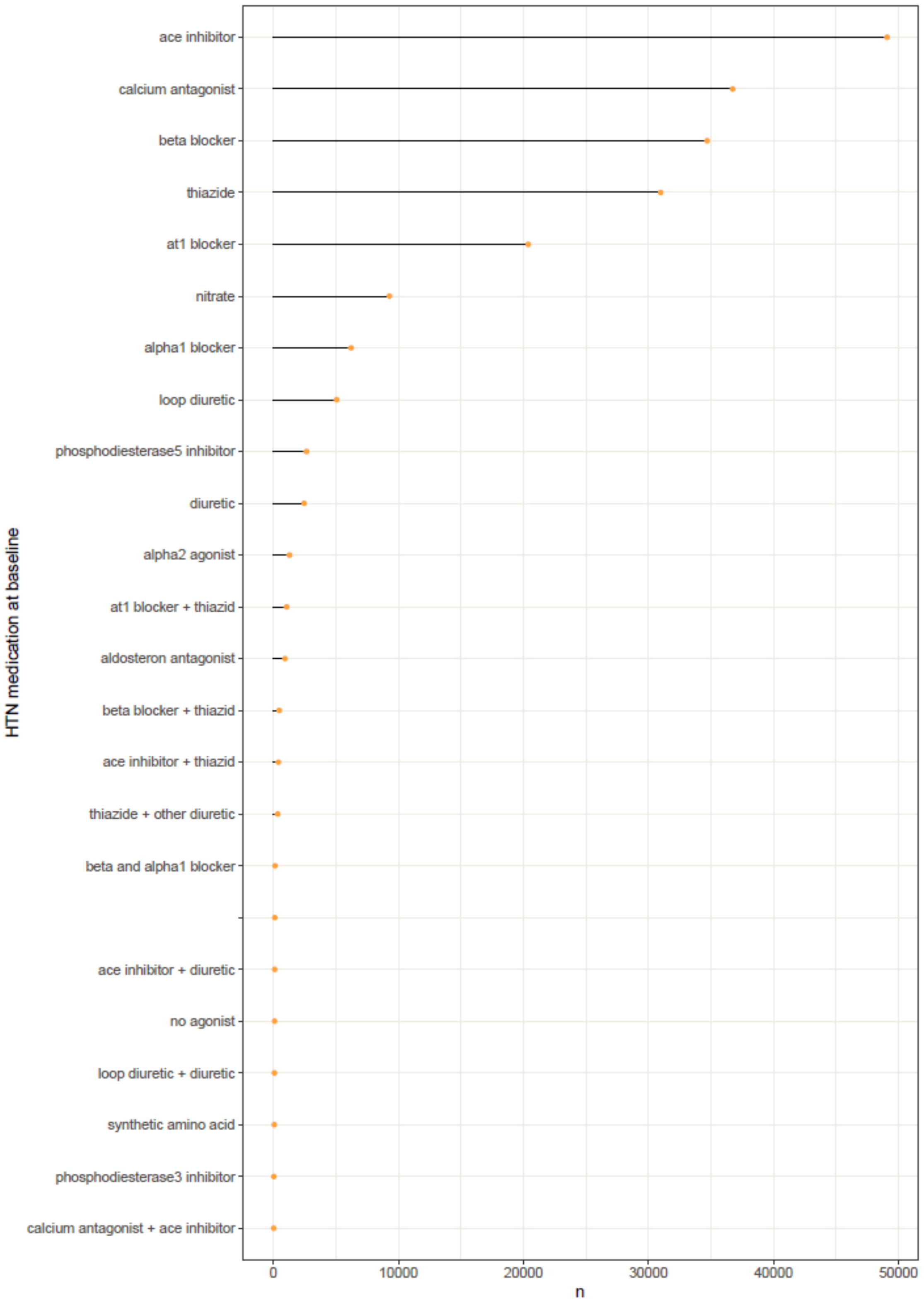

Supplementary Figure 2 – Frequencies of antihypertensive drug classes at baseline.

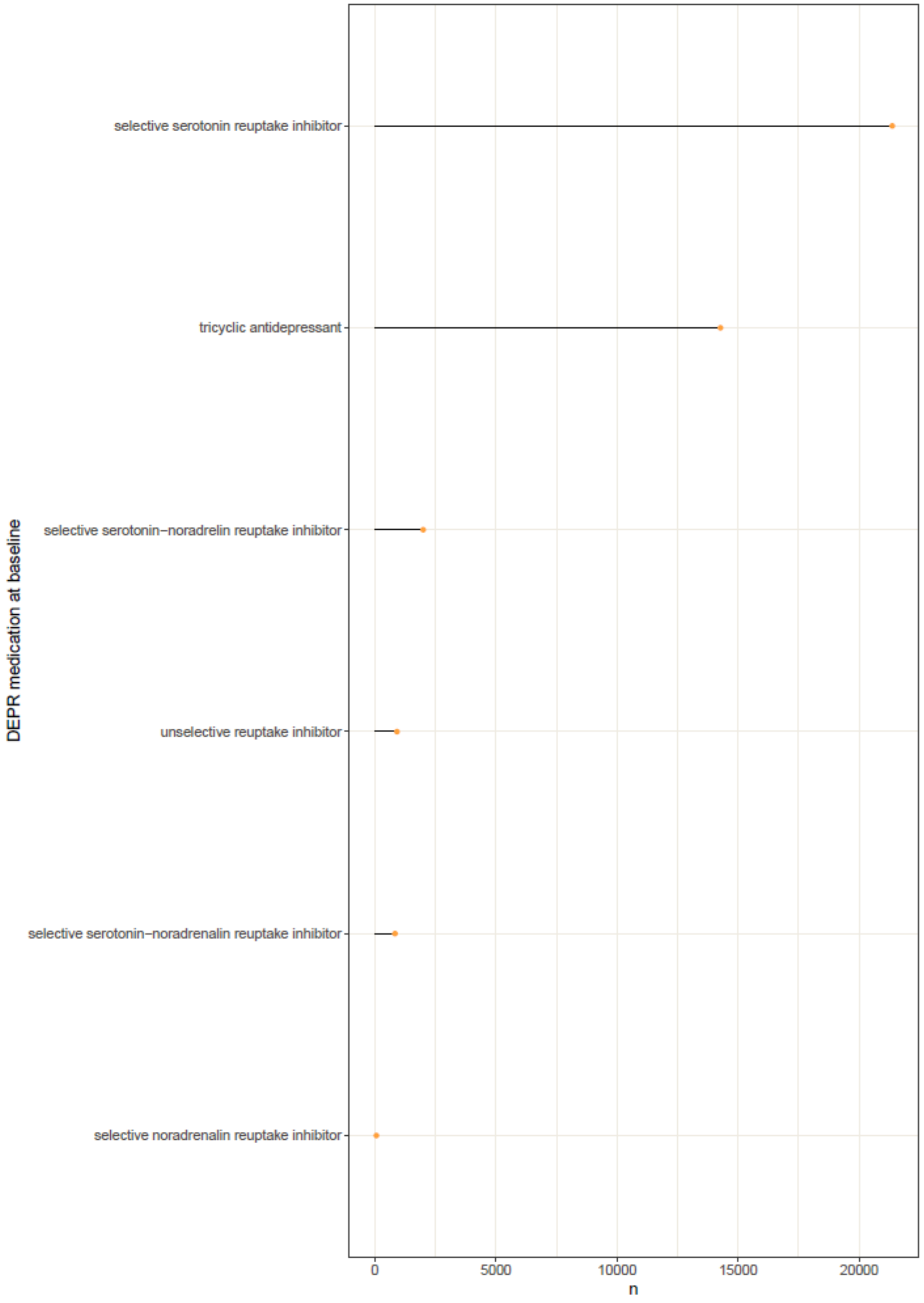

Supplementary Figure 3 – Frequencies of antidepressant drug classes at baseline.

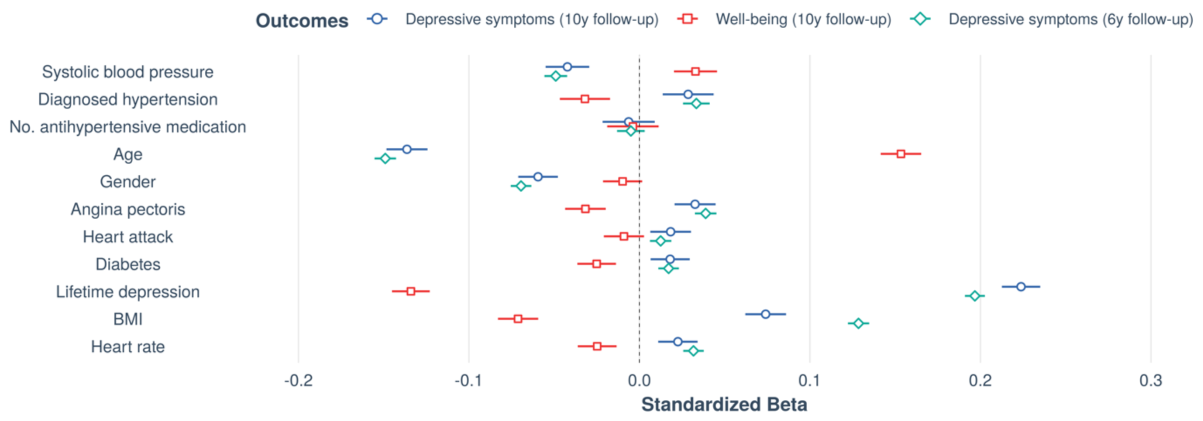

Supplementary Figure 4 – Online mental health follow-up results using the PHQ-9*. Longitudinal associations with mental health outcomes including PHQ-9 (i.e., "Depressive symptoms 6y follow-up") at follow-up assessment. Forest plot shows standardized beta estimates and 95% confidence intervals for predictors of interest (systolic blood pressure, diagnosed hypertension (HTN), and number of antihypertensives) as well as covariates at baseline.*

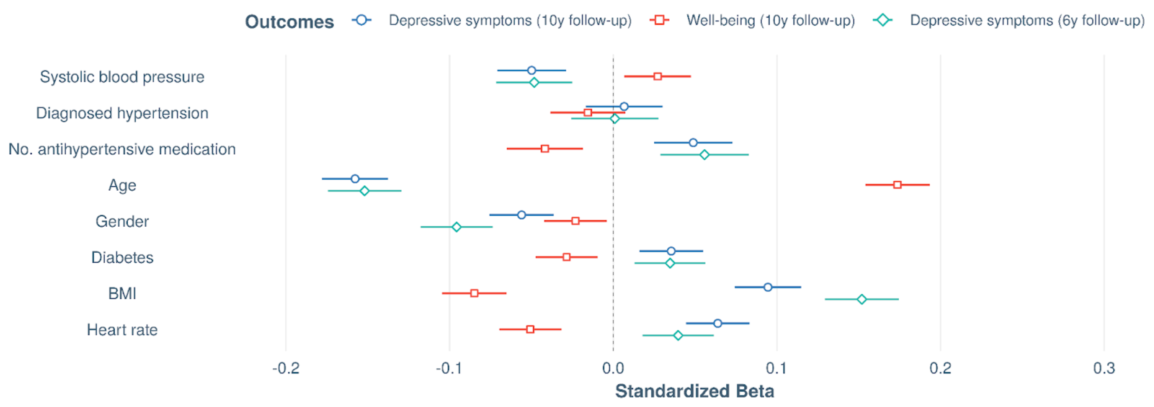

Supplementary Figure 5 – Cross-sectional associations of mental health outcomes at both follow-up assessments*. Forest plot shows standardized beta estimates and 95% confidence intervals for predictors of interest (systolic blood pressure, diagnosed hypertension (HTN), and number of antihypertensives) as well as covariates at 10-year follow-up. N = 10,333 for current depressive symptoms at 10-year follow-up, n = 8,122 participants with data for depressive symptoms at 6-year online mental health follow-up, and n = 10,990 participants with data for well-being at 10-year follow-up (after exclusion of missing values).*

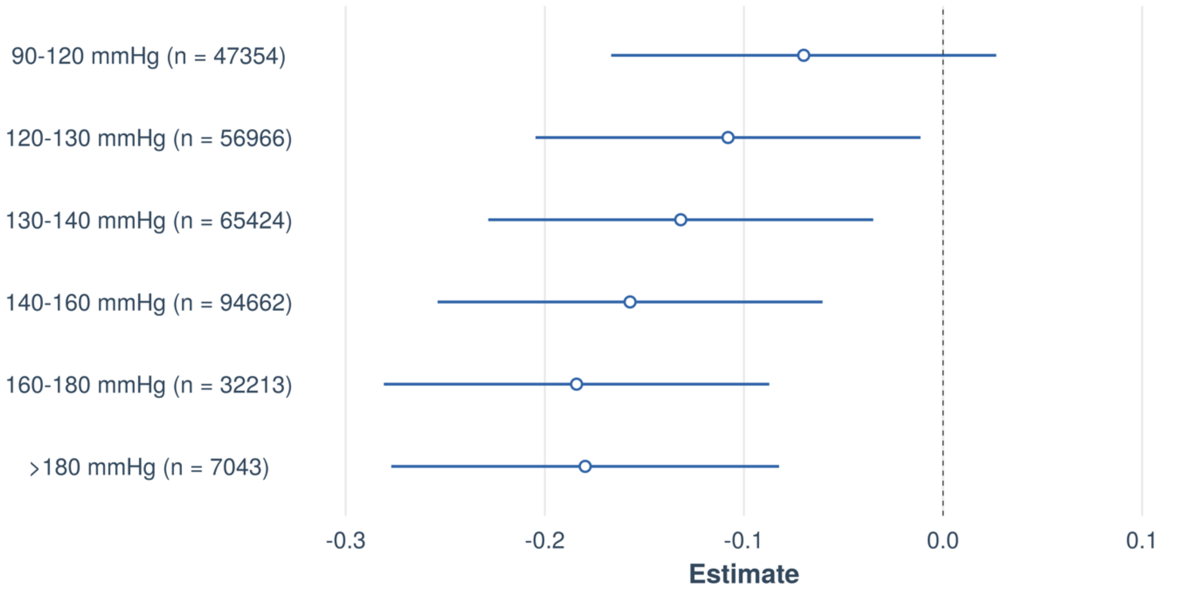

Supplementary Figure 6 – Association of depressive symptoms within systolic blood pressure categories. *Cross-sectional associations of depressive symptoms within systolic blood pressure categories at initial assessment. Forest plot shows regression estimates and 95% confidence intervals for each systolic blood pressure category and the respective sample size in each bin. SBP <90 mmHg (n = 109) served as the reference category in the model. The model has been fully adjusted for diagnosed hypertension, number of antihypertensives, and other covariates (age, gender, body mass index, resting heart rate, diabetes diagnosed by doctor (yes/no), lifetime depression diagnosed by doctor (yes/no), angina diagnosed by doctor (yes/no), myocardial infarction diagnosed by doctor (yes/no)). Total sample n = 303,771 participants (after exclusion of missing values).*

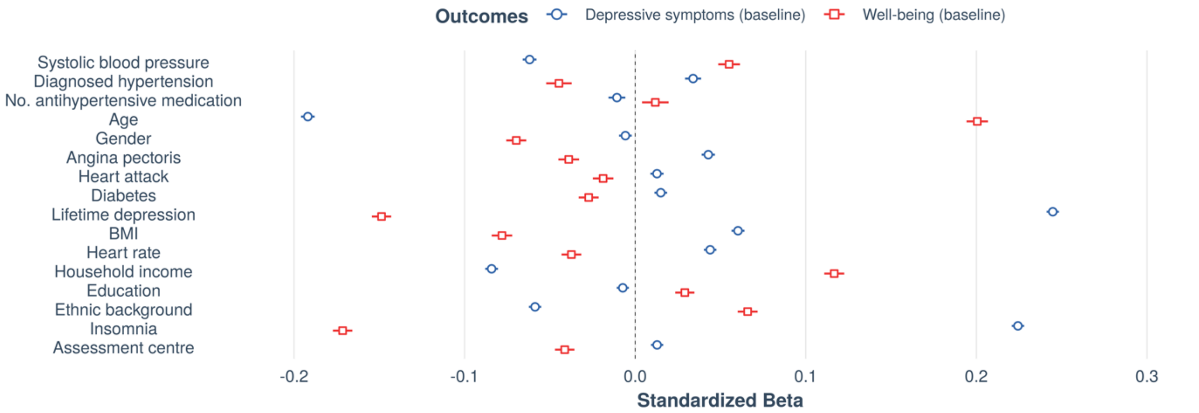

Supplementary Figure 7 – Modelling of additional relevant variables. *Cross-sectional associations with mental health outcomes including additional covariates (i.e., insomnia, assessment centre, household income, education and racial/ethnic background) at baseline assessment. Forest plot shows standardized beta estimates and 95% confidence intervals for predictors of interest (systolic blood pressure, diagnosed hypertension (HTN), and number of antihypertensives) as well as covariates at baseline.*

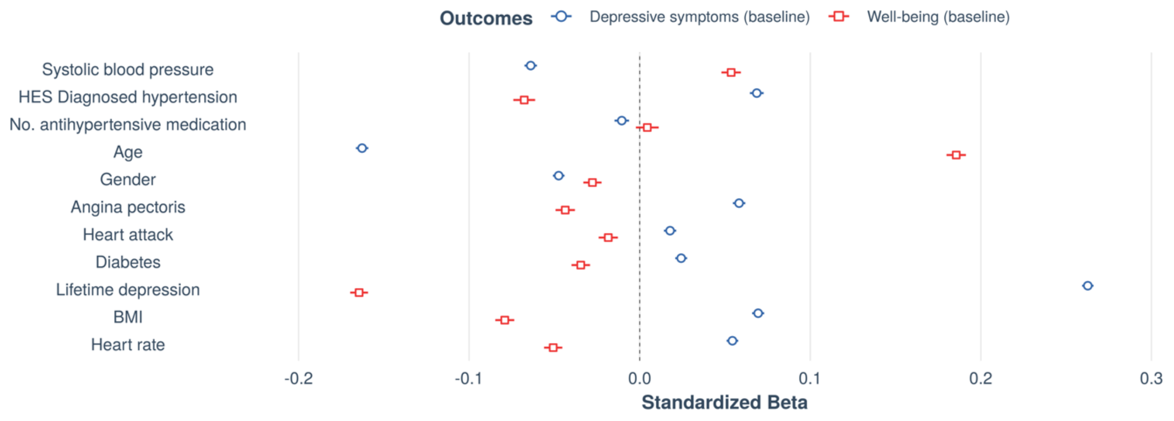

Supplementary Figure 8 – Analyses with hospital records (HES). *Cross-sectional associations with mental health outcomes including hospital-diagnosed HTN at baseline assessment. Forest plot shows standardized beta estimates and 95% confidence intervals for predictors of interest (systolic blood pressure, HES diagnosed hypertension, and number of antihypertensives) as well as covariates at baseline.*

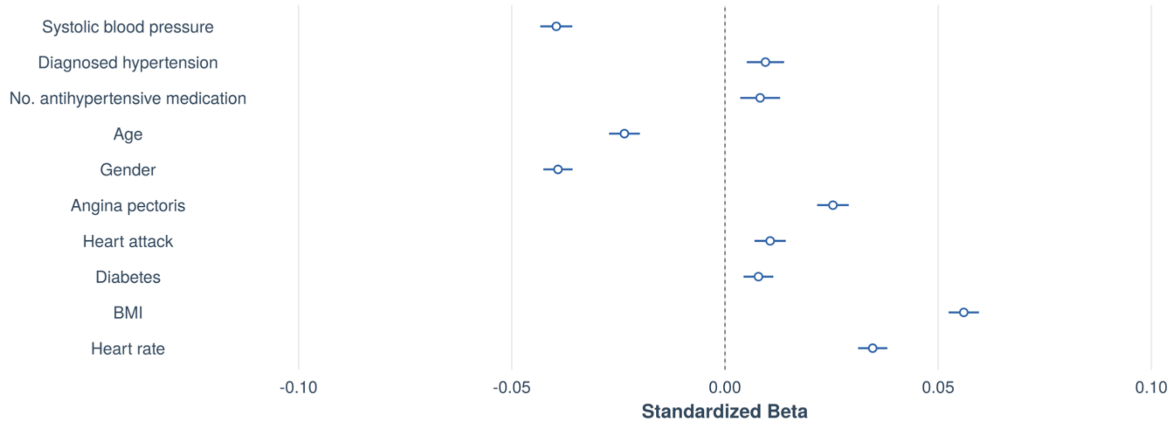

Supplementary Figure 9 – Analyses with hospital records (HES). *Cross-sectional associations with hospital-diagnosed depression as outcome. Forest plot shows standardized beta estimates and 95% confidence intervals for predictors of interest (systolic blood pressure, self-reported hypertension diagnosis, and number of antihypertensives) as well as covariates at baseline.*
