# Supplementary material for: Associations between mental health, blood pressure and the development of hypertension
Source: Nat Commun. 2023 Apr 7;14:1953. doi: 10.1038/s41467-023-37579-6 (PMC10082210; doi:10.1038/s41467-023-37579-6)
Supplement: Supplementary file 3 — Reporting Summary [file 41467_2023_37579_MOESM3_ESM.pdf]

nature portfolio

Corresponding author(s): Lina Schaare
Last updated by author(s): Jan 18, 2023

Reporting Summary

Nature Portfolio wishes to improve the reproducibility of the work that we publish. This form provides structure for consistency and transparency in reporting. For further information on Nature Portfolio policies, see our Editorial Policies and the Editorial Policy Checklist.

Statistics

For all statistical analyses, confirm that the following items are present in the figure legend, table legend, main text, or Methods section.

n/a	Confirmed	
☐	☒	The exact sample size (*n*) for each experimental group/condition, given as a discrete number and unit of measurement
☐	☒	A statement on whether measurements were taken from distinct samples or whether the same sample was measured repeatedly
☐	☒	The statistical test(s) used AND whether they are one- or two-sided<br>*Only common tests should be described solely by name; describe more complex techniques in the Methods section.*
☐	☒	A description of all covariates tested
☐	☒	A description of any assumptions or corrections, such as tests of normality and adjustment for multiple comparisons
☐	☒	A full description of the statistical parameters including central tendency (e.g. means) or other basic estimates (e.g. regression coefficient) AND variation (e.g. standard deviation) or associated estimates of uncertainty (e.g. confidence intervals)
☐	☒	For null hypothesis testing, the test statistic (e.g. *F*, *t*, *r*) with confidence intervals, effect sizes, degrees of freedom and *P* value noted<br>*Give P values as exact values whenever suitable.*
☒	☐	For Bayesian analysis, information on the choice of priors and Markov chain Monte Carlo settings
☒	☐	For hierarchical and complex designs, identification of the appropriate level for tests and full reporting of outcomes
☐	☒	Estimates of effect sizes (e.g. Cohen's *d*, Pearson's *r*), indicating how they were calculated

Our web collection on statistics for biologists contains articles on many of the points above.

Software and code

Policy information about availability of computer code

Data collection	This study did not collect any data. Data collection for UK Biobank data was carried out by the UK Biobank team.
Data analysis	All analyses and data visualisations were performed with R (4.0.2, R Core Team, 2015, Vienna, Austria; R-project.org/) and RStudio (version 2022.07.0), including R packages EValue (version 4.1.3), MICE (version 3.14.0), and lme4 (version 1.1-23). To allow for replication studies and reproducibility of our results, the analysis code can be found in the Open Science Framework repository of this study (https://osf.io/638jg).

For manuscripts utilizing custom algorithms or software that are central to the research but not yet described in published literature, software must be made available to editors and reviewers. We strongly encourage code deposition in a community repository (e.g. GitHub). See the Nature Portfolio guidelines for submitting code & software for further information.

Data

Policy information about availability of data

All manuscripts must include a data availability statement. This statement should provide the following information, where applicable:

- Accession codes, unique identifiers, or web links for publicly available datasets
- A description of any restrictions on data availability
- For clinical datasets or third party data, please ensure that the statement adheres to our policy

All data used in this study is available through the public resource of the UK Biobank (http://www.ukbiobank.ac.uk/). The raw UK Biobank data are protected and are not available due to data privacy laws. The UK Biobank data are available under restricted access to bona fide researchers for health-related research in the public interest. Access can be obtained by a registration and application to the UK Biobank resource. Data-field IDs of UKB variables used in this study are listed in the

Supplementary Material. The authors' access to the UK Biobank Resource was granted under Application Number 37721 (https://www.ukbiobank.ac.uk/enable-your-research/approved-research/understanding-the-role-of-the-brain-and-cardiovascular-factors-in-hypertension-affect-relationships).

Human research participants

Policy information about studies involving human research participants and Sex and Gender in Research.

Reporting on sex and gender	We refer to the variable gender and not sex, as the UK Biobank defines this variable as a mixture of the sex the NHS had recorded for the participant and self-reported sex, although we acknowledge that this does not capture the full spectrum of gender. We included gender as a covariate in all analyses, but did not include any gender-stratified analyses in this study.
Population characteristics	The sample, particularly the neuroimaging sub-sample, displays the healthy volunteer effect, which describes that UK Biobank participants are considered to be more health-conscious, self-reported fewer health conditions and show lower rates of all-cause mortality and total cancer incidence compared to the general population. Yet, associations between risk factors and disease outcomes in UK Biobank have been reported to be generalisable despite the healthy volunteer effect.
Recruitment	UK Biobank included participants from the UK between the ages of 40 and 69 years at recruitment in 2006 to 2010. Participants were contacted through postal invitations if they met the age range and lived within approximately 25 miles (40 km) of one of 22 assessment centers located throughout England, Wales, and Scotland. The size of the cohort was determined based on statistical power calculations for nested case-control studies to achieve large incidences and reliable odds ratios of common health-related conditions during the first years of the 20-years follow-up period.
Ethics oversight	The UK Biobank received ethical approval from the North West Multi-centre Research Ethics Committee (MREC; reference 11/NW/0382) in 2011, with renewed approval every 5 years since. This approval means that researchers do not require separate ethical clearance and can operate under the Research Tissue Bank (RTB) approval. All participants gave written informed consent.

Note that full information on the approval of the study protocol must also be provided in the manuscript.

Field-specific reporting

Please select the one below that is the best fit for your research. If you are not sure, read the appropriate sections before making your selection.

- [x] Life sciences
- [ ] Behavioural & social sciences
- [ ] Ecological, evolutionary & environmental sciences

For a reference copy of the document with all sections, see nature.com/documents/nr-reporting-summary-flat.pdf

Life sciences study design

All studies must disclose on these points even when the disclosure is negative.

Sample size	We used all available data in the UK Biobank for our measures of interest. The exact sample size for each analysis are reported in the main manuscript.
Data exclusions	No exclusion criteria were applied. In the exploratory moderation analysis, we excluded people with hypertension at baseline.
Replication	This is an observational study, reporting epidemiological associations observed in the dataset as a whole. Our results can be fully reproduced using the provided data analysis scripts in the Open Science Framework repository of this study (https://osf.io/638jg).
Randomization	There were no experimental groups in this study.
Blinding	Blinding was not necessary as there were no experimental groups in this study.

Reporting for specific materials, systems and methods

We require information from authors about some types of materials, experimental systems and methods used in many studies. Here, indicate whether each material, system or method listed is relevant to your study. If you are not sure if a list item applies to your research, read the appropriate section before selecting a response.

Materials & experimental systems

n/a	Involved in the study
☒	☐ Antibodies
☒	☐ Eukaryotic cell lines
☒	☐ Palaeontology and archaeology
☒	☐ Animals and other organisms
☒	☐ Clinical data
☒	☐ Dual use research of concern

Methods

n/a	Involved in the study
☒	☐ ChIP-seq
☒	☐ Flow cytometry
☒	☐ MRI-based neuroimaging
